# Cases of Fever in Children Treated by Leeches

**Published:** 1814-05

**Authors:** 


*For the Medical and Physical Journal.*

### *Cases of Fever in Children treated by Leeches.*

I WAS called to the infant daughter of Mr. B. residing in Tottenham-court-road, who was attacked the day before with symptoms of fever, viz. hot skin, florid countenance, thirst, sour breath; the tongue, nevertheless, was of a natural tural appearance. While in the room I observed her to start frequently, as if shivering with fits; this had also been remarked the preceding day, at the commencement of the attack, and she complained of cold, with pain in the head. Suspecting some mischief in the brain was going on, I desired to be called in case of any alteration for the worse, and ordered a saline aperient medicine to be given immediately. Before it could be administered, she was seized with a violent fit, which continued the space of half an hour. Two leeches were applied to the temples, but the convulsions had then abated, though the child was left in a state of insensibility. The leeches had scarcely filled, when an evident alteration had taken place for the better; the sensibility had returned, in the evening the fever was gone, and no appearance of convulsion has been since witnessed. On the following day, being still a little unwell, a piece of blistering plaster smeared on worsted was applied behind the ears, and by these means the whole of the complaint was removed.

Case II.—Mary, the infant daughter of A. T. of Hart-street, Bloomsbury, had been ill six months, and was observed to waste away without any manifest cause. About a month ago the ears became sore, and an eruption appeared on the surface of the body, with relief of the former indisposition, and it was generally remarked how much she was improved in health. The ears discharged until they became so painful as to produce considerable distress and irritation. Though not an advocate for the premature healing of these excoriations, having frequently traced hydrocephalic symptoms to this source, I advised an ointment, composed of calamine cerate and red precipitate, which in one day completely dried up the discharge. Our success, however, had nearly been attended with fatal consequences: fever, of a hectic kind, and headach, arose, with convulsive startings of the body; sometimes the countenance was flushed, and at others was of a deadly hue, with intervals of delirium. The friends of the child became seriously alarmed for its safety, and the whole college might have been summoned in consultation, had I not explained what I conceived to be the cause of the unfavourable symptoms, viz. *the healing of a discharge instituted by nature for the relief of the constitution.*

The antihumoral pathologist will probably smile at this opinion, though the fact which gives rise to it can neither be controverted or otherwise explained than upon this supposition. Leaving the theory, however, for the practice, leeches were applied with the same advantage in this case as in the former, but the symptoms were not entirely subdued dued until the mother of the child *suâ sponte* reproduced the ulceration, which continues to this day.

They who have witnessed the insidious approach of hydrocephalus, will be able to appreciate the importance of having recourse to local bleeding about the head in the fevers of children. I do not affirm that the cases here related are connected with this disease, but I have a distinct recollection of some fatal instances of it, where the first decidedly marked symptoms were exactly the same. I have learned from painful experience, in the person of a lovely child of my own, how difficult it is to detect its existence in the early stage, following the generally received accounts of the manner of its attack. As nothing can be more uncertain then than the first symptoms of hydrocephalus, it behoves the practitioner, in all cases where the possibility of doubt exists, to exercise extreme caution in the treatment of infantile complaints. X. Y.

*March 10th*, 1814.

